# Weather information improves a predictive model of emergency department arrivals

**DOI:** 10.1007/s11739-025-04062-7

**Published:** 2025-07-31

**Authors:** Athanasios Burlotos, Bryan A. Stenson, Anne V. Grossestreuer, Caleb Dresser

**Affiliations:** 1 Department of Emergency Medicine, Boston Medical Center, Boston, MA USA; 2 Department of Emergency Medicine, Beth Israel Deaconess Medical Center, Boston, MA USA; 3 Department of Environmental Health, Harvard T.H Chan School of Public Health, Boston, MA USA

**Keywords:** Operations, ED arrivals, ED volume, Weather, Environmental health, Climate change adaptation, Modeling

## Abstract

Variable emergency department (ED) volumes contribute to staffing challenges. Weather may impact ED arrival patterns. Enhancing prediction of arrivals based on weather conditions may be useful for operational planning, including adaptation to climate change. In this retrospective analysis of ED arrivals from an urban teaching hospital in the Northeast US from 2010 to 2019, two linear regression models were developed to predict daily arrival totals. The base model utilized calendar variables and a 28-day rolling average of daily arrival totals. The second model added weather variables. Models were tested for overfitting using training and test datasets, and residuals were plotted. Addition of weather variables to the model increased adjusted R-squared (0.418 to 0.483). Effect estimates and 95% confidence intervals for predictor variables in the final model were scaled to the mean daily arrival total (152.7) to facilitate interpretation. Monday was predictive of the most arrivals (+ 23.1%, CI + 22.0% to + 24.2%), relative to Sunday (reference). Holidays were associated with fewer arrivals (− 14.2%, CI − 15.9% to − 12.4%). Inclement weather was associated with fewer arrivals. Increases in the relative maximum daily temperature were associated with increases in arrivals. The effect of temperature varied across seasons. Inclusion of weather variables improved predictive value of a model of daily ED arrivals in this single-site, retrospective analysis. Rain, snow, and wind were associated with reduced arrivals, while warmer temperatures relative to historical averages were associated with increased arrivals. Inclusion of weather variables into models of ED utilization may support improved operational decision-making.

## Introduction

Emergency departments face escalating challenges related to overcrowding, boarding, staffing, and fluctuations in patient volume due to factors outside their control. Addressing these challenges is complex, and while many solutions lie outside the emergency department itself [1], there are opportunities to improve the operational response to these challenges. Improving understanding of the dynamics of emergency department arrivals, and ability to act on this knowledge, is one such opportunity.

ED arrivals represent the inflow of patients into the emergency department, and in many cases the hospital system. Fluctuations can lead to a mismatch between need and resources. Surges in arrival volume may lead to increased wait times, higher rates of patients who leave without being seen by a physician, and delays in care [2]. Low arrival volumes may lead to under-utilization of staff and resources. In both cases, anticipation of an unusually high or low volume of arrivals allows appropriate action to be taken ahead of time; this is seen in the routine upstaffing carried out in many emergency departments during known busy periods [3].

Previous operationally oriented studies of emergency department arrivals have focused on calendar-based variables, as well as the influence of isolated events such as sports games [4] and natural disasters, and larger global pandemics. [5] Emergency department administrators have frequently mapped arrival patterns by day of week and hour of day, as well as seasonal variability [6]. This research has shown typical peak arrivals between 10 AM and 10 PM, as well as higher volumes on Mondays. Understanding the ebbs and flows of patient arrivals has become increasingly necessary with the increased burden of emergency department crowding and its impact on the healthcare system [1].

In addition to these factors, weather conditions are likely to be of increasing importance to emergency medicine operations. Extreme weather events ranging from heat waves to severe storms are becoming more frequent and intense because of climate change [7]. Enhancing prediction of patient arrival volume in the context of weather conditions may be useful for operational adaptation to the impacts of climate change.

Previous research has demonstrated an association between emergency department utilization and a variety of weather and environmental factors. Studies have linked ambient temperature and emergency department utilization in a wide variety of settings and subpopulations. Temperature effects exist in the general population, among the elderly, among patients with dementia [8], among pediatric patients [9], among diabetics [10], and among people living with mental health conditions [11]. Studies of air pollution and wildfire smoke have identified relationships between concentrations of air pollutants and all-cause emergency department utilization [12, 13], as well as utilization for specific conditions, including respiratory and cardiovascular disease [14].

While it is clear there are relationships between emergency department utilization and weather and environmental factors, implications of these factors at the level of individual facilities from an operational standpoint are less well studied. It is difficult to downscale the findings of large-scale epidemiologic studies to specific operational environments, which are dependent upon both environmental conditions and effect modification by local geography, built environment, healthcare accessibility, population characteristics, and other unique local factors. Translation of epidemiologic knowledge into operationally actionable information calls for site-specific analyses. The goal of this project was to provide operationally relevant information about predicted emergency department volumes to inform staffing decisions and other resource planning. As a result, unlike traditional epidemiologic analysis, causal inference was not the primary objective. Instead, this study assessed the predictive value of adding weather information to a model of emergency department arrivals to support operational planning.

## Methods

### Study design

A retrospective, cross-sectional study was performed analyzing the association of various predictor variables with daily arrival totals in the emergency department of a single medical center. Specifically, linear modeling was used to study if there existed an association between weather conditions and daily arrival totals in the emergency department. This was done by generating linear regression models with and without the inclusion of weather variables and comparing their predictive value. The final model was run on both a training and test dataset to evaluate for overfitting.

### Ethics

This study was approved by the Committee on Clinical Investigations at Beth Israel Deaconess Medical Center (Protocol #2022P000002).

### Setting

This study was completed at an urban, academic medical center in the Northeast region of the United States with an annual census of roughly 55,000 patients per year. The study period included 10 years of data, from 2010 through 2019, and was selected to exceed the minimum sample size required for adequate power (*β* = 0.85) to detect (at *α* = 0.05) a small effect via multivariable regression with up to 15 variables, as well as availability of data. In addition, the study period was chosen to avoid the COVID-19 pandemic, during which emergency departments experienced unprecedented low volumes.

### Data sources

Records on hourly arrival totals from 2010 to 2019 were obtained from hospital records, which were then aggregated into daily totals. The hourly arrival volume nadir (6:00 AM) was used as the dividing point when aggregating daily totals, with arrivals prior to 6:00 AM being grouped with the preceding day. Federal holidays for the study period were obtained from published government historical records. In situations where an official holiday fell on a weekend, both the official holiday and the observed holiday (the Friday preceding or the Monday after) were counted as a holiday for the purpose of this study [15]. Historical daily weather records for the Boston Logan weather station (USW00014739) were obtained from the National Oceanographic and Atmospheric Administration (NOAA) [16]. Boston Logan was selected due to its close proximity to the study site (8.5 km), as well as the completeness of the records available during the study period. The 2020 U.S. Daily Climate Normals [17], which represent the 30-year average for daily temperatures for each day of the year, were also obtained for the same weather station.

### Selection and generation of variables

Variables from hospital records after aggregation included both the daily arrival total and the calendar date. The outcome variable for all portions of the analysis was the daily arrival total. Data joining, modification of variables, generation of new variables, and all statistical analysis were performed using the R statistical software [18–25]. Predictor variables for the analysis included both calendar factors or variables which were extracted from the calendar date and weather factors (which were obtained from the NOAA dataset). A summary of predictor variables is included below in Table 1. A 28-day rolling average term was calculated using the R package *RcppRoll*. [26] For the calendar factors, the day of the week was directly extracted from the calendar date using the *lubridate* package [27]. Variables related to the federal holidays were appended by comparing calendar date to the previously mentioned reference list of federal holidays. Days which corresponded to a federal holiday, or on which a federal holiday was celebrated, were marked with the binary variable “day of holiday”. Days for which the preceding day was a federal holiday (or a celebrated federal holiday) were marked with the binary variable “day after holiday”. A quantitative variable representing hours of sunlight was calculated using the R package *suncalc*, [28] based on the calendar date and GPS coordinates of the study site. Lastly, seasons were appended based on the meteorological convention.

**Table 1:** Summary and descriptive statistics of variables used in modeling. Characteristics include the percentage of days present. For continuous variables, the median and the range are also reported. Please note median and range calculations are based only on the days for which that variable has a recorded value (i.e., median mm of snow for days in which it snowed)

Variable	Source	Units	Characteristics
*Date*	index	–	[1/1/2010 to 12/30/2019]
*Arrival count*	Outcome	Arrivals per day	100% 154, [74 to 253]
*Day of week*	Calendar	The categorical day of the week (e.g., Monday)	100%
*Day of holiday*	Calendar	Holiday, yes or no	3.0%
*Day after holiday*	Calendar	Day which follows a holiday, yes or no	3.0%
*Sunlight*	Calendar	Hours of sunlight per day	100% 12.2, [9.1 to 15.3]
*Rolling average*	Calendar	Average arrivals per day, for the preceding 28 days	99.2% 154.0, [133.2 to 166.8]
*Precipitation*	Weather	Mm of precipitation, daily total	35.7% 3.8, [0.3 to 86.4]
*Snow*	Weather	Mm of snow, daily total	6.3% 23, [3 to 561]
*Wind average*	Weather	Average daily wind speed in m/s	100% 4.6, [1.0 to 17.0]
*Relative T*_*Max*_* spring*	Weather	℃ Above or below daily historical average, for spring days	25.2% − 0.2, [− 12.0 to 20.5]
*Relative T*_*Max*_* summer*	Weather	℃ Above or below daily historical average, for summer days	25.2% 0.6, [− 12.6 to 11.5]
*Relative T*_*Max*_* fall*	Weather	℃ Above or below daily historical average, for fall days	24.9% 0.1, [− 13.9 to 12.5]
*Relative T*_*Max*_* winter*	Weather	℃ Above or below daily historical average, for winter days	24.7% 0.0, [− 15.0 to 18.0]

Many possible weather variables were imported from the previously mentioned NOAA dataset. Initial selection of weather variables eligible for inclusion in the model focused on data completeness and avoidance of obvious collinearity (i.e., different measurements of the same phenomena). Similarly, variables for which recorded observations were extremely rare (< 1%) were also excluded. Given the many possible variables which are available in the NOAA dataset, discussion here will be restricted to a summary of important points. Full details of which variables were selected and why are available via review of comments in the annotated code files. Daily average wind speed was selected over other variations of wind speed measurements (such as peak wind speeds) given the arrival data was aggregated to daily totals. Average temperature, as well as humidity, were excluded as a possible variable in the weather model as it was not recorded for all years in the study period. Categorical, or binary, descriptions of the weather were excluded for two reasons. First, many of these categorical variables had positive entries for less than 1% of total study dates. Second, for the data with sufficient entries there was redundancy with quantitative variables (e.g., mm of rainfall versus presence of rainfall). Final weather variables included in the model are also highlighted below in Table 1.

### Model development

Model development proceeded in a stepwise process, beginning with an exploratory phase, followed by development of a calendar-based model, the addition of weather variables to the model, exploration of the effect of temperature, and lastly assessment of the final model. Exploratory data analysis included both data visualization and descriptive statistics. Data visualization was used to highlight overlying trends in the pattern of emergency department arrivals. One notable decision made on this visualization was the choice to add an hours of sunlight variable. Overall daily arrival patterns in aggregation were noted to follow a parabolic trend peaking in the summer months and with nadirs in December and January. Hours of sunlight was added to capture this variability as it reflects this observed pattern better than the categorical alternatives would have–for example adding season or month as a predictor variable.

Next, two linear regression models were developed. The base model utilized calendar variables (day of the week, day of a holiday, day after a holiday) and a 28-day rolling average of recent arrival volume. The decision to use a 28-day moving average is grounded in the operational (rather than epidemiologic) focus of this analysis. Unlike epidemiologic studies assessing the responsiveness of emergency department utilization to high temperatures, poor air quality, and other environmental health hazards, this study did not seek to provide causal inferences regarding their effects or to quantify the overall impacts of these hazards. Instead, this analysis was intended to improve upon existing operational approaches; a common assumption currently used is that recent events are a reasonable predictor of events in the near future. In this case, the use of a 28-day moving average helps account for seasonal variation in infectious diseases such as influenza, shifts in catchment area population related to academic calendars at local colleges and universities, changing socio-economic conditions, long-term changes in population during the study period, and other factors that are difficult to model and would be difficult to apply to a practical operational model even if they could be retrospectively obtained for this study.

Next, a second linear regression model was developed, which also incorporated weather variables. The exact variables included in both models are detailed previously, as is the initial selection process for which variables were retained. Final selection of variables in the models removed predictors that were not statistically significant, or which were weak predictors whose addition did not improve the model in a meaningful way. Given the operational focus of this project, a simpler model was favored over a more complex model which offered minimal improvements in performance. Prior to testing of the weather-informed model, further analysis was performed to determine which form of temperature optimized the performance of the model.

This was done based on a review of the literature, which showed that temperature is correlated with several health impacts as previously detailed; however, a convention of which form of temperature to use has not yet been established in the literature. Evaluation of which form of temperature would best fit the model was performed through a stepwise process. First, the previously mentioned multivariate calendar and weather informed model was developed, this model included all variables as detailed in Table 1, except for temperature. Next, the database was segmented according to meteorological seasons to allow for the effects of temperature on the model to be independently studied in each season. Then, temperature variables for each season were generated by generating combinations of the following axes: maximum daily temperature or minimum daily temperature, absolute temperature or temperature relative to historical normals, and linear relationships or transformed relationships were both explored. The resulting temperature variables were then incorporated into the previously mentioned weather-informed multivariate model, tested in each season, and the results on overall model predictive value were compared (via adjusted R-squared). Results showed that a linear (untransformed) relative maximum temperature performed best in all seasons except summer. In the summer, exponential transformations of relative maximum temperature outperformed the linear relationship. However, the improvement to overall model performance was minimal compared to the linear modeling. Therefore, untransformed (linear) relative maximum temperature was used for all seasons in the final model to simplify the model.

### Analysis

**Fig. 1:**
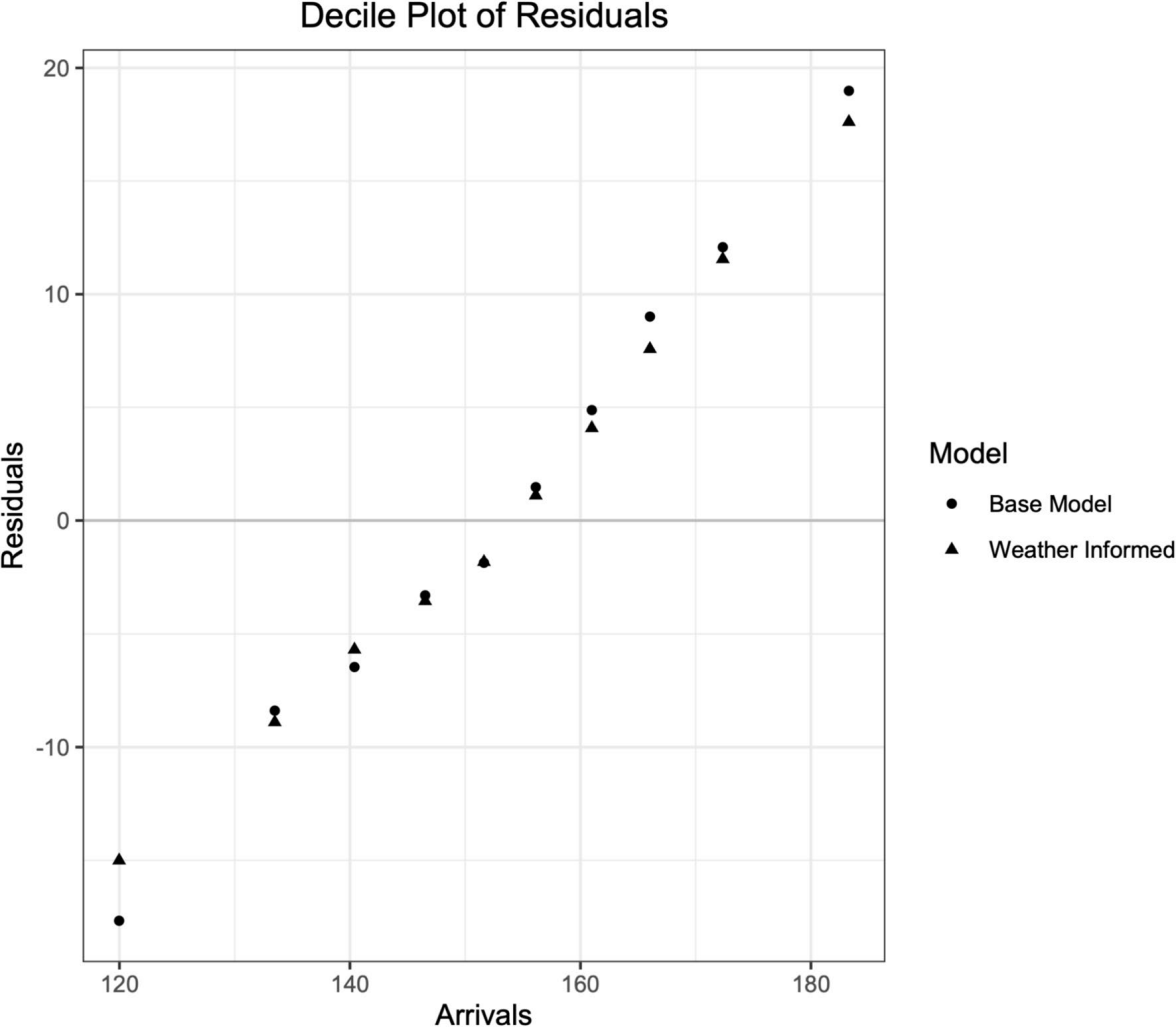
Decile plot of residuals from the test dataset for both the base model and weather informed model. Note that the model is biased toward the mean. The addition of weather variables to the model resulted in a moderate improvement. This plot highlights how this improvement manifested most prominently at the extremes of volume– the lowest and highest volume deciles

All modeling and analysis was done in the R Statistical Software using the base function *lm,* as well as the packages *dplyr*, *CaTools*, and *caret* [18, 19, 21, 22]. The performance of both the calendar and weather-informed model were compared using statistics of fit, specifically both unadjusted and multiple-testing adjusted *R*^2^. Significance of individual predictor variables was accessed using the p value resulting from linear regression modeling. Next, the final weather-informed model was tested for overfitting by segmenting the dataset into randomly selected training (80%) and test datasets (20%). The performance between the training and test models were compared using both unadjusted *R*^2^ and the significance of individual predictors. Statistical significance for individual predictors was defined with an alpha of 0.05. The effect sizes of individual predictor variables in the model were compared. This was done by first training the final weather-informed model on the entirety of the dataset. The 95% confidence interval for the absolute effect size (i.e., change in daily arrivals per unit of predictor variable) was then estimated using the linear regression model. To facilitate interpretation, these confidence intervals were then standardized to the percentage of the mean daily arrivals over the study period (i.e., percent increase or decrease in daily arrival volume relative to the average per unit of predictor variable). Lastly, residuals for the predicted versus observed daily arrival totals were calculated from the test dataset. These residuals were then aggregated into deciles and are presented in Fig. 1.

## Results

Data from 2010 to 2019 were obtained, for a total of 3,653 days of data, each with associated daily volume. Given the use of the 6 AM nadir as the division between days, the last day of the dataset was removed. This resulted in 3,651 days of data in the final dataset. The median daily volume was 154.0 arrivals. The distribution of arrivals was minimally skewed toward higher volume days, with an interquartile range of 26.0 (1st quartile 140.0, 3rd quartile 166.0). The minimum daily volume was 74, and the maximum daily volume was 233. Calendar-derived predictor variables were complete, with only the 28-day rolling average term having missing values, as there is a necessary lag in the calculation of this term. Daily weather data was obtained as described previously. There were no days with missing or unavailable weather data for the variables included in the final analysis.

The R-squared for the full dataset is reported as to highlight the overall explanatory value of the model, and because it can be adjusted for multiple testing. The full dataset was used to generate effect estimates for predictor variables as some variables (such as holidays) are rare events and the inclusion of all data allows for more accurate estimation of effect sizes. Segmented training and test dataset R-squared values are reported to evaluate the model for overfitting.

The base model relied on calendar variables including day of week, holiday, day after holiday, hours of sunlight, and a 28-day rolling average of emergency department arrivals. This model provided moderate explanatory value when tested using the full dataset (R-squared = 0.420, adjusted R-squared 0.418). Results were similar when using a training and test dataset to evaluate for overfitting (training dataset R-squared 0.423, test dataset R-squared 0.404). In application to the test set, residuals were distributed with positive slope. This signifies that the model was biased toward the mean. In other words, the model tended to over-predict emergency department arrivals on low-volume days and under-predicted arrivals on high volume days.

Addition of weather variables resulted in a modest increase in explanatory value when applied to the full dataset training set (R-squared = 0.485, adjusted R-squared 0.483). This improvement was also reflected when segmenting the dataset into training and testing datasets (training dataset R-squared 0.487, test dataset R-squared 0.473). With the addition of weather variables, the distribution of residuals was slightly improved. Specifically, inclusion of weather data improved model performance in the lowest and highest deciles of daily arrivals. Despite this, the model continued to demonstrate high residuals at extremes of volume. The residuals of both models are summarized in Fig. 1.

Effect estimates and 95% confidence intervals for predictor variables in the complete model were scaled to percentages of the mean daily arrival total (152.7) to facilitate interpretation. Calendar variables demonstrated large effect sizes; Mondays (+ 23.1%, CI + 22.0% to + 24.2%) and Fridays (+ 19.2%, CI + 18.1% to + 20.3%) were predictive of high arrival volume relative to Sundays (reference). Holidays were associated with fewer arrivals (− 14.2%, CI − 15.9% to − 12.4%); days following holidays demonstrated a small rebound effect (+ 6.1%, CI + 4.3% to + 7.9%).

Inclement weather was associated with fewer arrivals, as demonstrated by average daily wind speed (− 0.69% per km/h), precipitation (− 1.09% per cm of precipitation), and snow (− 0.89% per cm of snow). Increases in the maximum daily temperature relative to historical averages were associated with significant increases in arrivals in all seasons except the summer; however, the effect size varied significantly between the seasons. The increases in volume associated with maximum temperatures above historical averages were most prominent in the fall. Winter and spring also had significant arrival variation with temperature. Temperature was not independently a significant predictor in the summer. Effect estimates with confidence intervals from the complete model are presented in Table 2.

**Table 2:** Linear regression modeling results for predictor variables

Variable	Estimate	Units	Scaled estimate	Scaled 95% CI
Sunday	Reference			
Monday	35.2	arrivals per Monday	23.1%	[22.0%, 24.2%]
Tuesday	23.1	arrivals per Tuesday	15.1%	[14.0%, 16.3%]
Wednesday	20.7	arrivals per Wednesday	13.5%	[12.4%, 14.6%]
Thursday	20.2	arrivals per Thursday	13.3%	[12.2%, 14.4%]
Friday	29.3	arrivals per Friday	19.2%	[18.1%, 20.3%]
Saturday	8.4	arrivals per Saturday	5.5%	[4.4%, 6.6%]
Not holiday	Reference			
Day of holiday	− 21.6	arrivals per holiday	− 14.2%	[− 15.9%, − 12.4%]
Not day after holiday	Reference			
Day after holiday	9.3	Arrivals per day, for days which follow a holiday	6.1%	[4.3%, 7.9%]
Rolling average (28d)	0.56	Arrivals per day per average arrivals per day	0.37%	[0.31%, 0.42%]
Sunlight	0.52	Arrivals per day per hour of sunlight	0.34%	[0.18%, 0.50%]
Precipitation	− 1.66	Arrivals per day per cm of precipitation	− 1.09%	[− 1.48%, − 0.70%]
Snow	− 1.36	Arrivals per day per cm of snow	− 0.89%	[− 1.01%, − 0.77%]
Wind average	− 1.06	Arrivals per day per km/hr of wind	− 0.69%	[− 1.35%, − 0.04%]
Relative T_Max_ Spring	0.36	Arrivals per day per ℃ above daily historical averages	0.23%	[0.13%, 0.34%]
Relative T_Max_ Summer	0.16		0.11%	[− 0.03%, 0.25%]
Relative T_Max_ Fall	0.73		0.48%	[0.35%, 0.61%]
Relative T_Max_ Winter	0.38		0.25%	[0.14%, 0.36%]

Absolute estimates results are presented alongside scaled estimate results to facilitate interpretation. Scaled values were adjusted based on the mean daily arrivals across the study period (152.7)

## Discussion

The addition of weather variables to a model of emergency department arrivals improved explanatory value and predictive capabilities. While the complete model explains only about half of the variability in emergency department arrivals at this site, this is an improvement on a model based solely on calendar variables and recent arrival volume information.

Calendar based models are commonly used, both because of the relative simplicity of analyzing operational data via a limited number of categorical variables, and convenience of predictable calendar-based shift scheduling. However, these models have limitations, and addition of information on factors affecting patients’ health and behavior has the potential to improve prediction of surges or slow periods, with the potential to better align staffing with operational needs. In our model, addition of weather information resulted in an improvement in the multiple testing adjusted R-squared from 0.418 to 0.483. This could be interpreted as weather variables representing 6.5% of all variation in emergency department arrivals, independent of their correlation with calendar variables. From an operational standpoint, this represents a 16% relative improvement in modeling capability compared to a calendar-based model alone. While our complete model continues to under-predict the most extreme deviations from baseline arrival volume, it handles these better than a solely calendar-based model.

Predictor values in the model should be interpreted judiciously, and epidemiologic conclusions cannot be made based on this model. The use of a 28-day rolling average dampens the impact of weather factors substantially, as weather factors follow a seasonal pattern. If the rolling average term was removed from the model the effect sizes of the weather terms would increase substantially. We chose this approach because it was operationally useful, and our interest was in operationally significant findings and model simplicity, rather than epidemiologic analysis or causal inference.

However, even allowing for probable dampening related to the use of the rolling average term in the model, implications of certain weather events for anticipated emergency department arrivals at the study site were substantial. To appreciate this, it is important to understand that the weather variables display a high degree of collinearity. For example, a typical January winter storm at the study site might include the following weather variables: 20 cm of snow (7.9 inches), 2 cm of precipitation (0.8 inches, snow is also counted as precipitation via its water equivalent), average wind speeds of 20 km/hr (12.4mph), and temperatures 10 °C below historical averages for that day. Based on the modeling results described previously, the independent effect of weather would be predicted to be a 46% reduction in daily arrivals from the average day. Another example would be an atypically warm day in fall. The effect of a fall day 10 °C above historical averages would be an estimated 4.8% increase in daily arrivals.

The relationship that increasing temperatures result in increased emergency department arrivals is consistent with the existing epidemiologic literature cited previously. The overall trend of decreasing arrivals during inclement weather is aligned with the anecdotal experience of many emergency physicians. Seasonal variability was also substantial. While no causality can be inferred using our methodology, it would be plausible that some combination of health impacts from weather, choices about whether to seek care during inclement weather, transportation impacts, and unrelated variables—such as the presence or absence of college students in the hospital catchment area at various times of year—could contribute to overall volume trends on a daily or seasonal basis.

Understanding the factors influencing emergency department arrival volume will be increasingly important because of ongoing strains on emergency medicine and the escalating impacts of climate change. While anticipating periods of high or low emergency department arrivals will not eliminate boarding problems or solve staffing shortages, it will allow managers to make the best use of resources at hand. Solutions may range from up-staffing or down-staffing in anticipation of fluctuations in arrival volume to coordinating the transient opening of additional inpatient spaces.

Escalating intensity and variability of weather events including heatwaves, severe storms, and hurricanes resulting from ongoing anthropogenic climate change also have substantial implications for emergency department operations. Some facilities will be directly exposed to climate hazards; many will likely experience changes in emergency department presentations which may range from the effects of extreme heat to long-term changes in population related to the migration of people displaced by climate change. Modeling efforts such as those presented here are a step toward adapting to this new reality; their application for decision making support will require prospective validation in settings in which they are to be used.

## Limitations

Analysis was designed to provide an approach that can inform operational decisions and was not intended as an epidemiologic study; effect size estimates from the model should be interpreted with caution, as the presence of a 28-day moving average variable may distort the true magnitude of the effects of predictor variables relative to an analysis focused on causal attribution. Data are from a single center, and the model calibration described here is unique to that setting; similar models developed at other sites will likely show different relative predictive value for various factors, depending on characteristics of the population and built environment they serve. In addition, weather variables may have different impacts across regions. Weather data was obtained from a single weather station, which is not co-located with the hospital, and we are unable to account for spatial variability in weather exposure across the population served by this ED. Finally, arrivals were not stratified by ESI level or diagnosis, and it is possible some weather variables may have both positive and negative effects through different mechanisms; in our approach, we assess only the net effect on total arrival volume. It will be important to validate this model and modeling approach prospectively, particularly when applied outside the setting and region in which it was developed.

## Conclusion

Inclusion of weather variables improved predictive value and fit of a model of daily emergency department arrivals at the study site. Weather variables accounted for a moderate amount of the observed variation in emergency department arrivals in this single-site, retrospective analysis. Rain, wind, and snow were associated with reduced arrivals, while warmer temperatures were associated with increased arrivals. Inclusion of weather variables into models of emergency department utilization may support improved decision-making regarding resource allocation and have increasing value as climate change leads to increasing weather variability.

## Data Availability

Weather records used are publicly available through the U.S. National Oceanographic and Atmospheric Administration (NOAA). Hospital arrival data is confidential and not publicly available.
